# Functional brain responses to emotional faces after three to five weeks of intake of escitalopram in healthy individuals: a double-blind, placebo-controlled randomised study

**DOI:** 10.1038/s41598-024-51448-2

**Published:** 2024-02-07

**Authors:** Sophia Armand, Christelle Langley, Annette Johansen, Brice Ozenne, Oliver Overgaard-Hansen, Kristian Larsen, Peter Steen Jensen, Gitte Moos Knudsen, Barbara Jacquelyn Sahakian, Dea Siggard Stenbæk, Patrick MacDonald Fisher

**Affiliations:** 1 Neurobiology Research Unit, Copenhagen University Hospital Rigshospitalet, Copenhagen, Denmark; 2 Department of Psychology, Faculty of Social Sciences, University of Copenhagen, Copenhagen, Denmark; 3 Department of Psychiatry, University of Cambridge, Cambridge, UK; 4 Present Address: Department of Clinical Medicine, Faculty of Health and Medical Sciences, University of Copenhagen, Copenhagen, Denmark; 5 Section of Biostatistics, Department of Public Health, University of Copenhagen, Copenhagen, Denmark; 6 Department of Drug Design and Pharmacology, Faculty of Health and Medical Sciences, University of Copenhagen, Copenhagen, Denmark

**Keywords:** Cognitive neuroscience, Emotion, Pharmacology

## Abstract

Short-term intake of selective serotonin reuptake inhibitors (SSRIs) modulates threat-related amygdala responses in healthy individuals. However, how SSRI intake over a clinically relevant time period modulates threat-related amygdala responses is less clear. In a semi-randomised, double-blind, placebo-controlled study of 64 healthy individuals (SSRI n = 32, placebo n = 32), we examined the effect of 3–5 weeks of SSRI escitalopram (20 mg daily) on brain response to angry, fearful and neutral faces using BOLD fMRI. Data was analysed using a whole-brain region-wise approach extracting standardised effects (i.e., Cohen’s D). The study was conducted at the Copenhagen University Hospital. A priori, we hypothesised that SSRI would attenuate amygdala responses to angry and fearful faces but not to neutral ones. Whether SSRI modulates correlations between amygdala responses to emotional faces and negative mood states was also explored. Compared to placebo, 3–5 weeks of SSRI intake did not significantly affect the amygdala response to angry, fearful, or neutral faces (|Cohen’s D|< 0.2, *P*_FWER_ = 1). Whole-brain, region-wise analyses revealed significant differences in frontal (|Cohen’s D|< 0.6, *P*_FWER_ < .01) and occipital regions (|Cohen’s D|< 0.5, *P*_FWER_ < .01). SSRI did not modulate correlations between amygdala responses to emotional faces and negative mood states. Our findings indicate that a 3–5 week SSRI intake impacts cortical responses to emotional stimuli, an effect possibly involved in SSRI’s therapeutic efficacy.

*Trial registration* Clinical Trials NCT04239339.

## Introduction

Affective cognition in humans describes the cognitive processing of emotional information in the environment^1,2^. Facial expressions represent a universal and rich source of emotional information^3,4^, with threat-related expressions such as angry or fearful facial expressions being important cross-cultural emotional cues that guide human behaviour to, for example, avoid danger^1,2^. Meta-analyses have established a functional brain atlas of emotional face processing, including visual, limbic, temporal-parietal, and prefrontal areas, the putamen, the cerebellum, and the amygdala^5^. Of these regions, the amygdala is particularly important for detecting salience and social relevance of emotional stimuli^5–8^, especially for threat-related salience^2,9–12^. Notably, there is evidence to suggest that some degree of activity in the amygdala in response to threat is adaptive^13–15^, but that hyperactive amygdala reactivity to threat is maladaptive and involved in Major Depressive Disorder (MDD)^16–19^.

Serotonin (5-HT) is a neural signalling system that shapes various behavioural phenotypes^20,21^, including affective cognition across healthy and clinical populations^22–30^. The 5-HT transporter (5-HTT) is a protein critically involved in controlling the duration and magnitude of 5-HT signalling as it clears 5-HT from the extracellular space^31^. The 5-HTT is the main target of Selective serotonin reuptake inhibitors (SSRIs), a group of drugs prescribed as the first-line pharmacological treatment of MDD. Clinically, SSRIs are administered to attenuate depressive symptomology, such as negative mood states, with clinical effects typically evident after at least two weeks^32^. Research suggests that these clinical effects of SSRI are mediated by changes in affective cognition, for example, by attenuating amygdala response to threat^33,34^. However, this was not supported in a recent study in MDD^35^. Investigating how SSRI intake over a clinically relevant time impacts brain response to threat in healthy individuals can elucidate potential treatment mechanisms of SSRIs. However, such investigations are sparse, and whether longer-term SSRI intake modulates threat-related brain responses in healthy individuals remains unclear.

Combining SSRI administration with functional Magnetic Resonance Imaging (fMRI) is a strategy to investigate serotonergic modulation of relevant brain function. fMRI studies of healthy individuals evaluating the effects of acute (i.e., within hours) and short-term (i.e., 7–10 days) intake of SSRIs compared to placebo have generally reported an altered amygdala response to emotional stimuli^36–39^, with the majority reporting reduced amygdala response to threat^40–45^. Effects of short-term SSRI intake on emotional face processing have also been observed in other brain regions, for example, in the frontal cortex^46^. Only two studies (one conducted in our lab) have investigated how SSRI intake over a clinically relevant time, i.e., more than two weeks^32^, modulates amygdala response to emotion in 13 females and 32 males, respectively^24,47^. These two studies did not find an overall effect of SSRI intake on amygdala response to emotions. However, sub-analyses in one study revealed that compliant females (n = 10), as verified by sufficiently high SSRI urine concentrations, displayed reduced amygdala response to threat compared to placebo^47^. Examining the effects of SSRI intake on emotion processing in the brain over a clinically relevant time frame would benefit from including a protocol with continuous reminders and verifications for compliance. Also, including a relatively larger sample of healthy individuals with an equal sex distribution is important as MDD is prevalent in both males and females, and they show different clinical responses to SSRIs^48^. It further remains to be investigated how amygdala responses to emotion after SSRI intake over a clinically relevant time correlate with mood states.

In 64 healthy individuals, we evaluated the effects of 3–5 weeks of intake of the most selective SSRI, escitalopram, versus placebo on amygdala response to angry, fearful, and neutral faces using fMRI in a double-blinded study with a rigorous compliance protocol. We included neutral faces to examine whether sub-chronic SSRI selectively modulates amygdala response to negative emotions or facial expressions per se. We hypothesised that the amygdala response to angry and fearful facial expressions would be lower but unchanged to neutral faces in response to SSRI compared to placebo. Additionally, we evaluated the effects of SSRI on region-wise whole-brain responses to facial expressions. Finally, we explored whether 3–5 weeks of intake of SSRI modulated the correlation between amygdala responses to facial expressions and negative mood states in our healthy sample.

## Methods and material

### Ethics

All experimental protocols were approved by the Danish ethics committee for the capital region of Copenhagen, Denmark (i.e., De Videnskabsetiske Komiteer, Region Hovedstaden) with journal ID H-18038352, and with amendments numbers 71579, 73632 and 78565. All methods were performed in accordance with relevant guidelines and regulations including the Declaration of Helsinki. Participants were informed about the study, including potential side effects and risks. Informed consent was obtained from all participants. The study was preregistered at ClinicalTrials.gov (Identifier: NCT04239339) on the 27/01/2020.

### Participants

**Figure 1:**
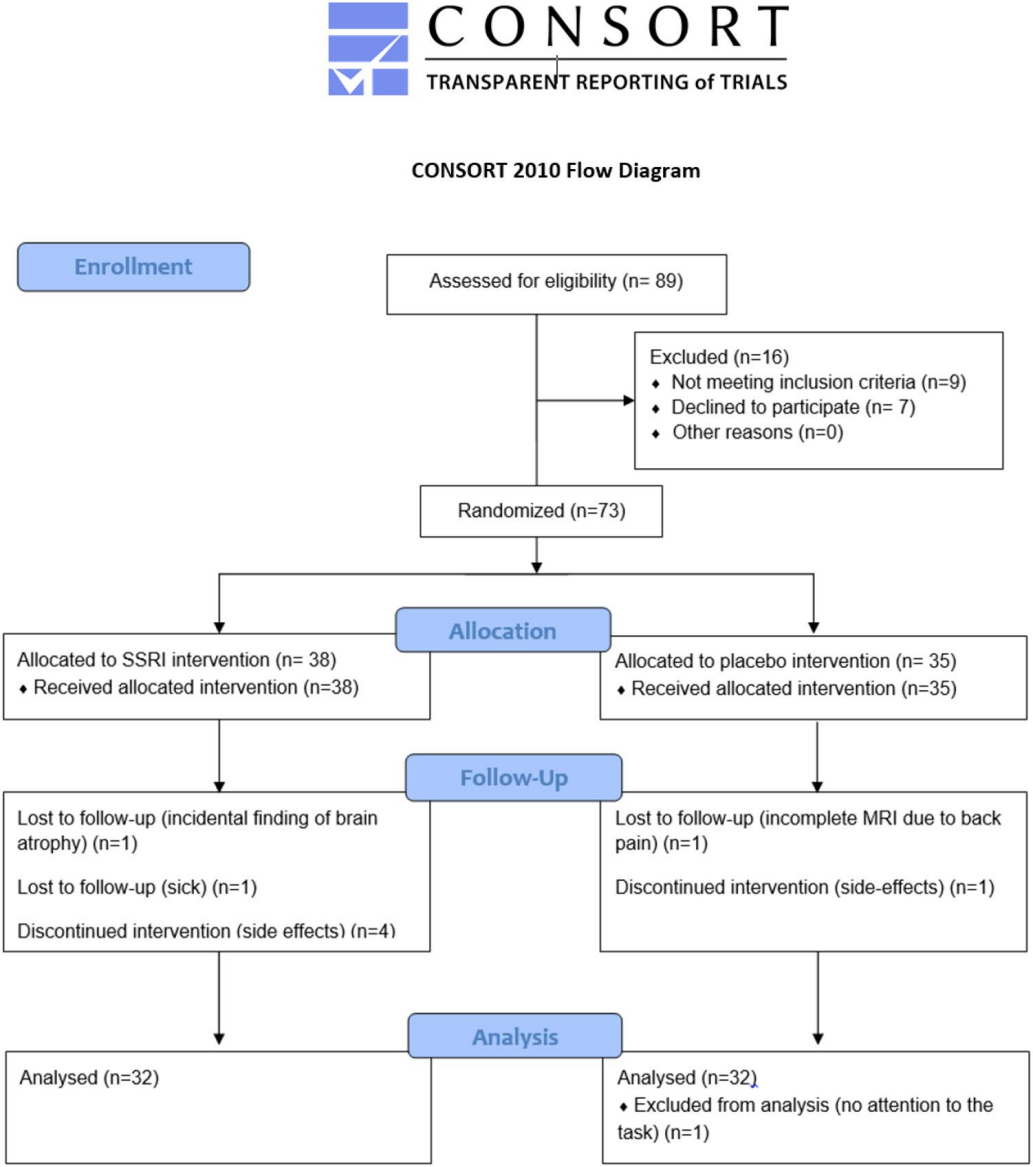
Consort Flow Diagram presenting the number of research volunteers enrolled, allocated to intervention, completed the study to follow-up and were included in the data analysis.

In total, 64 healthy volunteers (mean age in years (SD) [range]: 25 (6) [19–45], 41 females) completed the study. The volunteers were recruited from a list of individuals who expressed interest in participating in brain research. See Fig. 1 for the CONSORT flow diagram of the inclusion of research volunteers and reasons for missing data, which is detailed in appendix 1 in the supplementary material. The participants underwent screening for somatic illness, including a medical examination, blood screening for somatic disease, an ECG, and the presence of psychiatric conditions. See appendix 2 in the supplementary material for the exclusion criteria. The primary outcome of the overall study was to assess effects of SSRI on cognition assessed behaviourally, which is published elsewhere^49^. The primary outcomes were used to determine the study’s sample size, detailed in the aforementioned publication.

### Experimental design and drug administration

In a double-blinded design, participants were semi-randomised stratified with respect to sex, age, and IQ to receive a clinically relevant dose of escitalopram (20 mg daily in capsules of 10 mg)^50^ or placebo in identical capsules for 3–5 weeks, representing a clinically relevant time period of SSRI intake^32^. The randomisation was done by an administrative staff member uninvolved in inclusion, data collection or analysis. Capsules were manufactured and distributed by the Capital Region Pharmacy in Denmark. Participants received oral and written instructions for taking escitalopram, including possible side effects. They were instructed to take one capsule (10 mg escitalopram in the active group) each morning for 3 days and two capsules (20 mg escitalopram in the active group) daily on the fourth day and until the last day of examination after 3–5 weeks. The study was conducted at the Copenhagen University Hospital, Rigshospitalet, between May 2020 and October 2021.

Participants were asked to fill in a daily medication logbook, recording the time and number of capsules taken, serving as a daily reminder to take the capsules and providing additional information about participant compliance. A blood sample was taken halfway through the intervention period and again at follow-up, typically in the morning before MRI, to verify intervention compliance. Further, participants' capsule containers were inspected at each visit, and their medication logbook was verified at the follow-up visit. Participants were instructed to take the capsule after the blood sample on blood sampling days. During the intervention period, side effects were monitored weekly. If participants experienced unusual side effects or other complications during the study period, they could contact the project physician.

Following the intervention period, participants completed an MRI scan session. Before MRI scanning, participants completed self-report questionnaires to evaluate their psychological state. Participants and investigators involved in data acquisition and analysis were blinded to intervention type until completion of data analysis. Once participants had completed the study, we assessed the blinding efficacy by asking whether they believed they had received an active drug or placebo.

### Study measures

#### Psychometrics

Before participants commenced the intervention, we measured their intelligence quotient (IQ) using the Reynolds Intellectual Screening Test^51^, body mass index (BMI) and administered questionnaires to assess levels of depressive symptoms using the Beck's Depression Inventory II (BDI-II) (range: 0–63, where a score above 20 indicates a dysphoric or depressed mood)^52^, levels of state anxiety using the State-Trait Anxiety Inventory (STAI)^53,54^ (range: 0–80, higher levels indicating more anxiety, but no cut-off adapted to indicate severe clinical anxiety) and stress level using the Cohen's Perceived Stress Scale (PSS) (range: 0–40, higher levels indicating more stress, but no cut-off adapted to indicate severe clinical stress)^55^.

Both at baseline and follow-up (i.e., on the same day as MRI scanning), and typically in the morning, we assessed participants' level of negative mood states using the validated self-report questionnaire, the Profile of Mood States (POMS)^56,57^. The questionnaire was developed to measure transient mood states from 65 items using a 5-point Likert scale ranging from 0 (not at all) to 4 (extremely), from which a total composite score indicates the level of negative mood states (i.e., tension/anxiety, depression/dejection, anger/hostility, fatigue/inertia, and confusion/bewilderment versus vigour/activity). Total score ranges from − 32 to 200, with higher scores indicating a more negative or disturbed mood but no cut-off indicative of severe mood disturbance^57^.

#### Emotional faces fMRI paradigm

The emotional faces paradigm consisted of four blocks of emotional faces (i.e., fear, anger, surprise, and neutral blocks, presented in a randomised order) interleaved with five control blocks of geometric shapes (i.e., circles and vertical/horizontal ellipses) and lasted 6.5 min^35^. A trio of stimuli was presented, with two on the bottom of the screen and one on the top. Participants were instructed to identify which of the two stimuli on the bottom matched the one on top as quickly and accurately as possible. For the faces blocks, a trio of stimuli were presented, including two similar faces to match and one odd face, all expressing the same emotion. The matching task was intended to ensure that participants attended to the stimuli. Written instructions (i.e., "match faces") were displayed (2 s) at the beginning of each faces block. Within each face block, six face trios were presented for four seconds each, interleaved with a fixation cross (" + ") presented for two, four or six seconds, mean = 4 s, to minimise expectancy effects and habituation. The control block followed a similar procedure; written instructions (i.e., "match shapes") were displayed for two seconds, and the fixation cross was displayed for fixed 2-s periods. The paradigm and participants’ accuracy and reaction times were recorded in E-prime (Psychological Software Tools, Pittsburgh, USA). See appendix 3 for details on MRI acquisition. As we were only interested in the effects of SSRI on amygdala response to negative and neutral faces, we only included and analysed data from the angry, fearful and neutral face conditions, as done in previous studies^23,24,29,58^. The emotional faces paradigm was a secondary outcome of the overall study.

### Data analysis

#### Pre-processing and analyses of fMRI data

See appendix 4 for a description of pre-processing of fMRI data. We analysed data from each participant in general linear models (GLMs) by employing a canonical hemodynamic response function to the smoothed functional images to estimate task-specific BOLD activity (i.e., beta images). A high-pass filter (128 s) was applied to control for slow-frequency fluctuations. Motion parameters and censored volumes were included as covariates. The GLMs were used to generate contrast images (linear combination of task-specific effects) for our effect of interest (i.e., fearful, angry, and neutral faces contrasted to geometric shapes). Next, group-level analyses were used to determine population-level brain responses to fear, anger, and neutral faces in the SSRI group compared to the placebo group. The amygdala, our primary ROI, and all other brain areas were defined using the WFU PickAtlas v3.0.3^59,60^. For each participant, we extracted mean signal values from the bilateral amygdala for the main analyses, and for hemisphere-specific, whole-brain regions for secondary analyses, which were evaluated further in R version 4.0.3 (https://cran.r-project.org/).

We also conducted exploratory post hoc voxel-wise analyses to probe possible sub-regional effects. Following pre-processing that involved warping data in Montreal Neurological Institute (MNI) space (see appendix 4 for details), we fit the same GLMs described above to estimate task-specific BOLD responses for each participant, which were used in a group-level design matrices to estimate intervention effects.

#### Quantification of serum escitalopram concentration

We analysed the concentration of serum escitalopram (nmol/L) using ultra-high performance liquid chromatography/tandem mass spectrometry (Filadelfia Epilepsy Hospital, Dianalund, Denmark).

#### Statistical analysis

We included data for angry, fearful, and neutral faces and geometric shapes from the emotional faces paradigm. The data was analysed using a block design. Blocks of geometric shapes were collapsed and used to contrast each facial expression (e.g., BOLD response to angry faces versus BOLD response to shapes). We used linear regression models to evaluate differences in the amygdala response to angry, fearful, and neutral faces between SSRI and placebo groups. Age and sex were included as covariates. Using the same model, we performed analyses of group differences in whole-brain regional responses to angry, fearful, and neutral faces. To visualise these effects, we display regional standardised effects (Cohen’s D) of SSRI mapped onto the set of voxels belonging to each respective region for a brain image in standard MNI space. As post-hoc analyses, we used the same statistical model to identify group differences on a voxel-wise level.

We used linear regression models to evaluate whether amygdala responses to each emotional face were correlated with negative mood states, as measured with POMS on the same day, and whether SSRI modulated such potential correlation using an interaction term between amygdala responses and group. Age and sex were included as covariates. We computed post-hoc group-wise partial correlations between emotional amygdala responses and negative mood states with age and sex as covariates.

Across all tests, *P*-values are reported uncorrected (*P*_unc_) and corrected for family-wise error rate (*P*_FWER_) using the Bonferroni correction method. For the a priori amygdala analyses, we controlled for a family of three tests and the whole-brain analyses for a family of 88 tests. *P*-values were considered significant at *P*_FWER_ < 0.05. Effects sizes are reported as Cohen’s D. For the voxel-wise analysis, we used the AFNI-based 3dClustSim and 3dFWHMx functions to determine a cluster size unlikely to occur by chance at an uncorrected voxel-level threshold of *P* < 0.001, that maintains a type-I error rate of 0.05. For whole-brain and amygdala ROI-specific analyses, this was determined to be k > 211 and 2 voxels, respectively.

We used one-sample t-tests, including the whole sample, to examine whether the emotional faces paradigm induced a significant response in the amygdala. We used chi-square or two-sample t-tests to evaluate any differences between the groups on baseline measures, including sex, age, IQ, BMI, BDI-II, STAI, PSS and POMS, as well as POMS, censored volumes during fMRI, and participants’ blinding at follow-up. For the emotional faces paradigm, accuracy rates were positively skewed, so we evaluated group differences using a two-sample permutation test with 10,000 Monte Carlo replications for each emotion. Group differences in reaction time for each emotion were assessed using t-tests. *P*-values are reported uncorrected (*P*_unc_).

## Results

### Sample characteristics

Participant demographics, psychometrics, serum escitalopram levels, and emotional amygdala responses as assessed with the emotional faces paradigm and its behavioural outcomes are reported in Table 1. BDI scores confirmed the absence of dysphoric or depressed mood (i.e., < 20; score range: active group = 0–9, placebo group = 0–12)^52^. STAI and PSS levels across groups were aligned with typical scores in a healthy cohort (STAI, score range: active group = 20–39, placebo group = 20–36;^44^ PSS score range: active group = 2–20, placebo group = 1–19)^52^. The participants' mean IQ was in the normal upper range, i.e., higher than the average 100 IQ point of the general population, across both the active group (mean ± SD = 110 ± 8) and the placebo group (mean ± SD = 108 ± 7). Overall, there were no significant differences in baseline characteristics between the escitalopram and placebo groups (see Table 1 for group-wise descriptive data and estimate and *P*-values from statistical tests of group differences).

**Table 1:** Descriptive information for participants in each group along with group differences.

Baseline						
	Placebo group (N = 32)		SSRI group (N = 32)		Group dif	
	Frequency	Percent	Frequency	Percent	Est.	Punc
Female/male	20/12	63%/37%	21/11	66%/34%	1.12	0.29

	Mean (SD)	Median [Min, Max]	Mean (SD)	Median [Min, Max]	Est.	Punc
Age (years)	25.8 (5.9)	23.9 [19.9, 45.6]	24.8 (5.6)	22.7 [19.3, 41.9]	0.2	0.49
IQ	108 (6.9)	110 [90.0, 118]	110 (8.07)	107 [97.0, 129]	− 0.3	0.27
BMI (kg/m2)	24.0 (3.9)	23.1 [18.9, 40.3]	23.9 (3.8)	23.1 [17.5, 32.8]	0.0	0.87
Days of intervention	25.8 (2.8)	26.0 [21.0, 36.0]	26.1 (2.8)	25.0 [22.0, 33.0]	− 0.1	0.56
Depression (BDI-II)	3.2 (3.3)	2.5 [0, 12.0]	2.4 (2.6)	2.00 [0, 9.0]	− 0.1	0.29
State anxiety (STAI)	25.0 (4.5)	24.0 [20.0, 36.0]	24.7 (4.1)	23.0 [20.0, 39.0]	0.1	0.82
Stress (PSS)	9.03 (4.4)	9.00 [1.00, 19.0]	8.3 (3.9)	8.00 [2.00, 20.0]	0.2	0.47
Mood dis. (POMS)	1.5 (12.2)	− 0.5 [− 14.0, 39.0]	− 0.7 (10.4)	− 2.00 [− 17.0, 32.0]	0.1	0.44

Mid-intervention						
	Mean (SD)	Median [Min, Max]	Mean (SD)	Median [Min, Max]	Est.	Punc
Serum esci. (nmol/L)	< 10 (−)	< 10 [−]	82.9 (59.6)	69.0 [28.0, 338]	–	–

Post-intervention						
	Placebo group (N = 32)		SSRI group (N = 32)		Group diff	
	Frequency	Percent	Frequency	Percent	Est	Punc
Guess (ssri/placebo)	5/26*	16%/81%*	17/15	53%/47%	4.13	0.04

	Mean (SD)	Median [Min, Max]	Mean (SD)	Median [Min, Max]	Est.	Punc
Mood dis. (POMS)	8.1 (22.2)	1 [− 23.0, 81.0]	3.7 (15.4)	3.5 [− 17.0, 58.0]	0.2	0.37
Serum esci. (nmol/L)	< 10 (−)	< 10 [−]	82.7 (49.8)	67.5 [28.0, 263]	−	−

	Mean (SD)	Median [Min, Max]	Mean (SD)	Median [Min, Max]	Est.	Punc
Amyg. angry	0.38 (0.74)	0.40 [− 1.4, 2.8]	0.29 (0.81)	0.15 [− 1.09, 2.42]	0.1	0.63
Amyg. fearful	0.31 (0.51)	0.35 [− 0.83, 1.31]	0.33 (0.47)	0.37 [− 0.71, 1.30]	− 0.1	0.68
Amyg. neutral	0.28 (0.61)	0.29 [− 1.03, 1.37]	0.39 (0.74)	0.31 [− 0.92, 2.66]	− 0.2	0.59
Acc. for angry (%)	0.97 (0.08)	1.00 [0.67, 1.00]	0.98 (0.05)	1.00 [0.83, 1.00]	− 0.2	0.78
Acc. for fearful (%)	0.97 (0.08)	1.00 [0.67, 1.00]	0.97 (0.06)	1.00 [0.83, 1.00]	0.0	1.00
Acc. for neutral (%)	0.98 (0.05)	1.00 [0.83, 1.00]	0.98 (0.06)	1.00 [0.83, 1.00]	0.0	0.95
Acc. for shapes (%)	0.96 (0.06)	0.97 [0.73, 1.00]	0.93 (0.1)	0.97 [0.73, 1.00]	0.3	0.61
RT for angry (ms)	909 (174)	870 [639, 1330]	888 (173)	849 [519, 1330]	0.1	0.64
RT for fearful (ms)	861 (166)	852 [622, 1260]	833 (184)	785 [553, 1260]	0.2	0.52
RT for neutral (ms)	875 (159)	874 [650, 1390]	856 (147)	809 [599, 1150]	0.1	0.62
RT for shapes (ms)	817 (120)	799 [591, 1030]	805 (135)	791 [561, 1140]	0.1	0.71

Group differences in age, IQ, BMI, BDI-II, STAI, PSS, and POMS both at baseline and follow-up as well as reaction times in the emotional faces paradigm, were assessed using two-sample t-tests with estimate representing Cohen's D. Group differences in sex and blinding were assessed using the chi-square test with the estimate representing χ2. As accuracy rates were positively skewed, group differences were assessed using a two-sample permutation test with 10,000 Monte Carlo replications for each emotion, with the estimate representing Cohen's D. Group differences in amygdala response to angry, fearful and neutral faces were assessed using linear regression models with age and sex as covariates with estimate representing Cohen's D. *One missing. *Abbreviations* PSS, Cohen’s Perceived Stress Scale; BDI-II, Beck Depression Index II; POMS, Profile of Mood States; ms, milliseconds; SD, standard deviation; IQ, intelligence quotient; BMI, body mass index; SSRI, selective serotonin reuptake inhibitors administered 3–5 weeks; Est., estimate, Punc, uncorrected *P*-value; diff., difference; dis., disturbance; esci., escitalopram; amyg., amygdala response to; acc., accuracy; RT, reaction time.

### Treatment compliance and assessment of blinding

The serum escitalopram concentrations, as measured approximately halfway through the intervention period (mean ± SD: 12 ± 2 days) and at follow-up (mean ± SD: 26 ± 3 days), confirmed that all participants in the escitalopram group complied with the drug intervention. Although the fraction of participants in the escitalopram group who guessed that they got escitalopram was at a chance level (53%), the number was significantly higher than in the placebo group (χ^2^ = 4.13, df = 1, *P*_unc_ = 0.04). See Table 1 for descriptive data and estimate and *P*-values from statistical tests of group differences.

### Amygdala responses to the emotional faces paradigm

Across the placebo and escitalopram groups, we observed a significant response of the amygdala to angry (mean ± SD: 0.333 ± 0.772, *P*_unc_ = 0.001), fearful (mean ± SD: 0.316 ± 0.479, *P*_unc_ < 0.001), and neutral faces (mean ± SD: 0.332 ± 0.670, *P*_unc_ < 0.001) relative to geometric shapes. There was no evidence of significant differences between groups in accuracy or reaction time (see Table 1). Further, there were no significant group differences in censored volumes (*P*_unc_ = 0.81).

**Figure 2:**
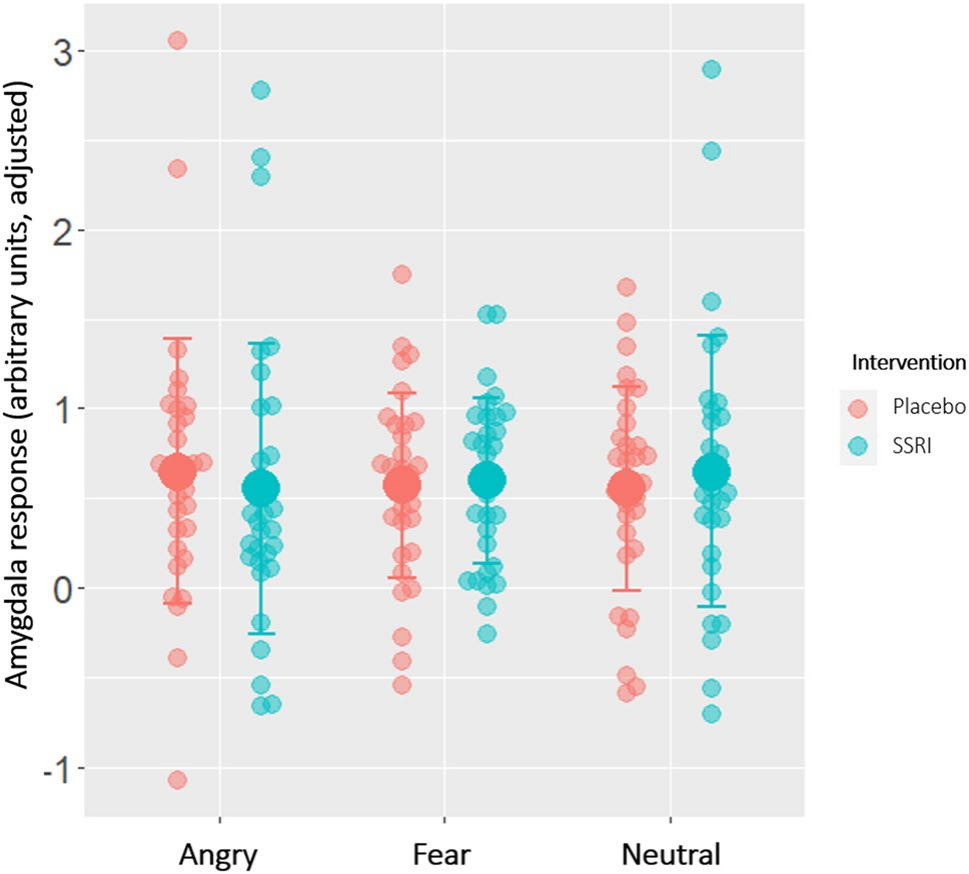
Bilateral amygdala response to angry, fearful and neutral faces (contrasted with geometric shapes) by group (SSRI, n = 32; placebo, n = 32), adjusted for effects of age and sex. Linear regression models revealed no significant differences between groups on amygdala response to emotional faces (angry: Cohen’s D = 0.1, *P*_FWER_ = 1.00; fear: Cohen’s D = − 0.1, *P*_FWER_ = 1.00; neutral: Cohen’s D = − 0.2, *P*_FWER_ = 1.00). Smaller transparent circles denote individual observed values and larger circles and bars denote sample mean ± 1 SD.

We observed no statistically significant difference between the SSRI and placebo group on amygdala response to angry, fearful or neutral faces (angry: mean difference = − 0.10, SE = 0.47, *P*_FWER_ = 1.00; fear: mean difference = − 0.05, SE = 0.12, *P*_FWER_ = 1.00; neural: mean difference = − 0.09, SE = 0.17, *P*_FWER_ = 1.00) relative to geometric shapes. Evaluation of sex differences showed that, across treatment groups, males had a significantly higher amygdala response to fearful faces than females (mean difference = 0.33, SE = 0.12, *P*_FWER_ = 0.02). Otherwise, no sex differences were observed (angry: mean difference = − 0.01, SE = 0.21, *P*_FWER_ = 1.00; neutral: mean difference = − 3.0, SE = 0.17, *P*_FWER_ = 0.26). Effects of age were not observed either (angry: mean difference = − 0.01, SE = 0.02, *P*_FWER_ = 1.00; fear: mean difference = 0.00, SE = 0.01, *P*_FWER_ = 1.00; neutral: mean difference = − 0.01, SE = 0.01, *P*_FWER_ = 1.00). See Table 1 for group-wise descriptive data as well as Cohen’s D and *P*-values from the statistical test of group differences, Table S1 in the supplementary material for detailed estimates and corrected *P*-values from the statistical models assessing the effect of escitalopram on amygdala response to emotional faces, and Fig. 2 for individual and average amygdala responses to each emotion adjusting for the effect of sex and age.

### Whole-brain responses to the emotional faces paradigm

**Figure 3:**
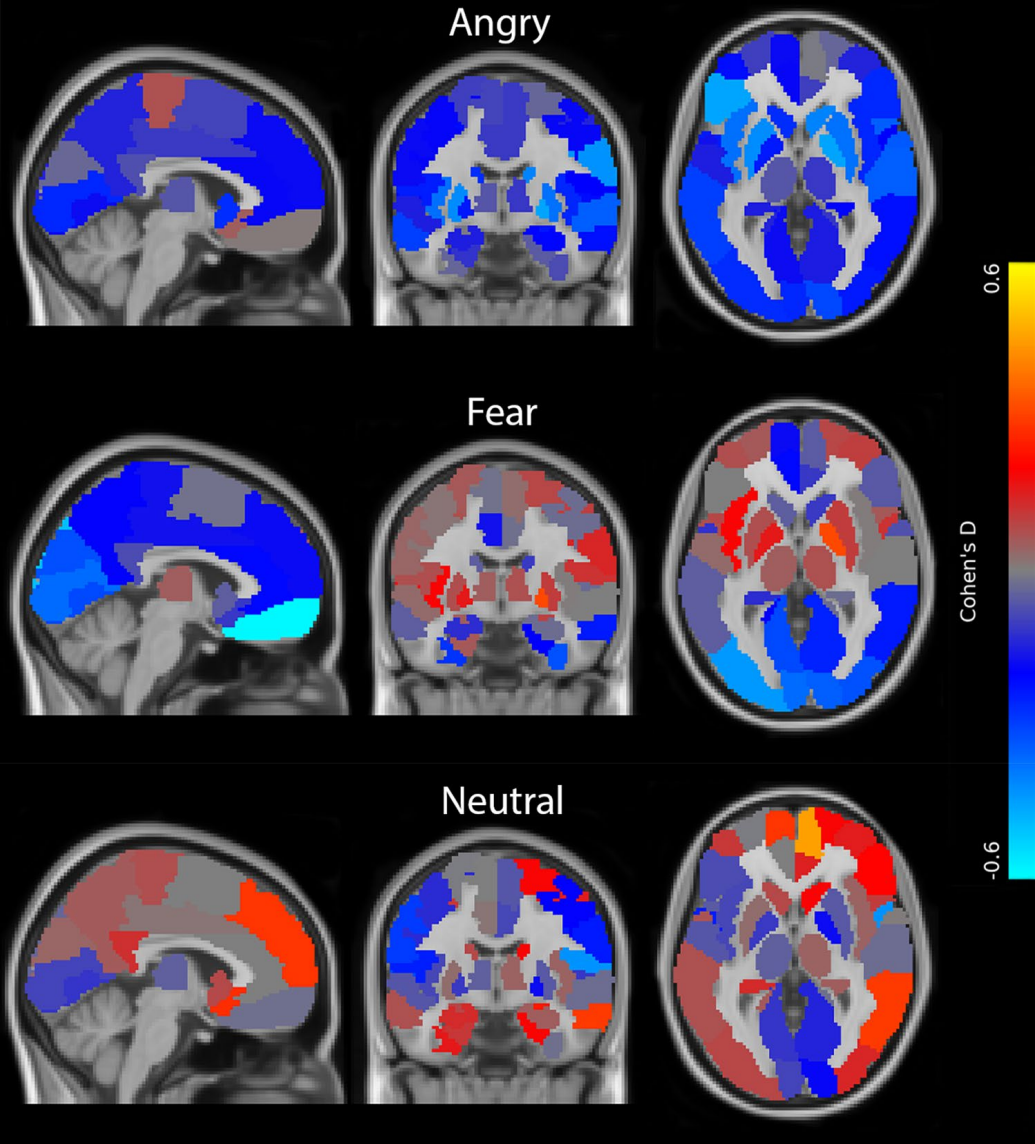
Illustration of standardised effect sizes (i.e., Cohen’s D) for regional effects of SSRI vs placebo on brain responses to emotional faces (i.e., angry, fearful and neutral faces constrasted with geometric shapes) in 64 healthy individuals. The regional Cohen’s D is displayed for each voxel within each of the 90 AAL regions, i.e., cortical and subcortical regions. Blue colours indicate negative values, i.e., lower response in the SSRI group, and red/yellow colours indicate positive values, i.e., higher response in the SSRI group. FSLeyes coordinates of brain slices across emotions: y = − 8.3, x = − 3.6, z = 0.7.

Analyses of the effects of 3–5 weeks of intake of escitalopram on whole-brain regional responses revealed that responses to angry faces were significantly lower in the left opercular part of the inferior frontal gyrus (mean difference = − 0.25, SE = 0.05, *P*_FWER_ < 0.001), in the left triangular part of inferior frontal gyrus (mean difference = − 0.21, SE = 0.06, *P*_FWER_ = 0.025) and in the right pallidum (mean difference = − 0.16, SE = 0.04, *P*_FWER_ = 0.025) in the escitalopram group compared to placebo. For fearful faces, the responses were significantly lower in the left gyrus rectus (mean difference = − 0.25, SE = 0.05, *P*_FWER_ < 0.001), in the left superior occipital gyrus (mean difference = − 0.26, SE = 0.07, *P*_FWER_ = 0.008), in the left middle occipital gyrus (mean difference = − 0.20, SE = 0.06, *P*_FWER_ = 0.04) and in the right inferior occipital gyrus (mean difference = − 0.31, SE = 0.08, *P*_FWER_ = 0.004) in the escitalopram group compared to the placebo group. For neutral faces, the responses were significantly higher in the right orbital part of the inferior frontal gyrus (mean difference = 0.27, SE = 0.06, *P*_FWER_ = 0.004) and in the right medial part of the superior frontal gyrus (mean difference = 0.23, SE = 0.06, *P*_FWER_ = 0.027). See Fig. 3 for an illustration of the whole-brain region-wise effects of SSRI. See Table S3, S4, and S5 in the supplementary material for region-wise estimates and *P*-values from the statistical models.

### Exploratory voxel-wise responses to the emotional faces paradigm

Across the whole-brain and within the amygdala, we did not observe any clusters showing a statistically significant effect of the SSRI intervention.

### Negative mood states

There was no significant group difference in negative mood states at baseline or follow-up (see Table 1). We found no significant correlations between amygdala responses to angry, fearful, or neutral faces and negative mood states in the placebo group (angry, *P*_FWER_ = 0.33; fear, *P*_FWER_ = 0.12; neutral, *P*_FWER_ = 0.07) or for a modulatory effect of escitalopram (angry, *P*_FWER_ = 0.49; fear, *P*_FWER_ = 0.29; neutral, *P*_FWER_ = 0.19). For an overview of the results, see Table 2. See Table S2 in the supplementary material for an overview of all group-wise partial correlations.

**Table 2:** The models explore whether emotional amygdala responses is correlated with negative mood states and whether this correlation is modulated by 3–5 weeks' SSRI intake, controlled for covariates age and sex.

	Estimate	SE	*P*_unc_	*P*_FWER_
Estimated model coefficients when regressing amygdala response to angry and negative mood states				
Constant	13.93	11.35	0.225	
Amygdala response to angry	7.24	4.47	0.111	0.332
SSRI	− 1.64	5.03	0.746	1.000
Amygdala response to angry*SSRI	− 8.63	6.12	0.164	0.493
Age	− 0.18	0.41	0.659	1.000
Sex	− 10.82	4.86	0.030	0.090

Estimated model coefficients when regressing amygdala response to fear and negative mood states				
Constant	18.41	10.82	0.094	
Amygdala response to fear	− 14.15	6.71	0.039	0.118
SSRI	− 9.60	5.39	0.080	0.241
Amygdala response to fear*SSRI	16.27	9.65	0.097	0.292
Age	− 0.08	0.41	0.836	1.000
Sex	− 10.78	4.96	0.034	0.102

Estimated model coefficients when regressing amygdala response to neutral and negative mood states				
Constant	6.47	12.00	0.592	
Amygdala response to neutral	13.45	5.70	0.022	0.065
SSRI	− 0.89	5.10	0.863	1.000
Amygdala response to neutral*SSRI	− 13.18	7.30	0.076	0.189
Age	0.07	0.42	0.864	1.000
Sex	− 10.96	4.74	0.024	0.073

Sex is the marginal effect of men relative to women.

The amygdala response to angry, fearful and neutral faces (contrasted with geometric shapes) are assesed using BOLD fMRI. Mood disturbances are measured with the self-report questionnaire Profile of Mood States (POMS), with higher values indicating more disturbance. Estimate is the unstandardised beta. *Abbreviations* SE B, standard error for unstandardised beta; Punc, unadjusted significance level; SSRI, selective serotonin reuptake inhibitors. All *P*-values were corrected for family-wise multiple testing using the Bonferroni method (i.e. all *P*-values multiplied by three), displayed under PFWER.

## Discussion

In 64 healthy individuals, we evaluated functional brain responses to angry, fearful, and neutral faces following 3–5 weeks of intake of either the SSRI escitalopram or placebo with a rigorous compliance protocol. This constitutes the largest such study to date in healthy individuals. Data was analysed using a whole-brain region-wise approach extracting standardised effects (i.e., Cohen’s D). Compared to placebo, SSRI did not significantly affect amygdala responses to faces (relative to geometric shapes), a finding inconsistent with our hypothesis. However, whole-brain, region-wise analyses identified frontal and occipital brain regions wherein the response to negative faces (i.e., anger and fear) was lower but higher to neutral faces following SSRI intake compared to placebo. Our findings contrast with evidence for acute and short-term SSRI intake changing the amygdala response to emotional faces in healthy individuals but implicate that SSRI intake over a clinically relevant period modulates a broader set of brain regions, including the frontal and occipital cortex. The valence-specific reduction of response to negative emotions in cortical brain regions following SSRI may be involved in the clinical efficacy of SSRI.

A primary region of interest in our analyses was the amygdala considering previous related studies^24,47^. We observed a pronounced and significant amygdala response to the emotional faces paradigm and a high degree of accuracy across both the SSRI and placebo groups, comparable to previous reports^8,35^. As such, the lack of a significant effect of SSRI on amygdala reactivity during emotion processing does not appear to stem from a weak or atypical neural response to the emotional faces paradigm. Two previous studies evaluating SSRI effects over a similar time frame also report a limited impact on the amygdala response to threat-related or happy faces^24,47^. However, in a sub-analysis, one of the previous studies reported that SSRI reduced amygdala response to threat in treatment-compliant participants, as confirmed by urinary drug concentration^47^. The authors suggested that the lack of a significant group difference could be attributed to non-compliance with the treatment. In our study, all participants were compliant with the treatment verified via serum SSRI concentration and self-report daily medication logbook, which suggests that non-compliance is not the cause of the non-significant results observed in our current study. Taken together, current evidence does not support that SSRI intake over a clinically relevant time period significantly modulates amygdala response to emotional faces across healthy populations. Future questions to be resolved include whether SSRI intake over a clinically relevant time period impacts other emotional processes involved in MDD, such as reinforcement sensitivity^62^.

Our whole-brain, region-wise analyses showed significantly lower brain responses to angry and fearful faces following SSRI intake compared to placebo in cortical regions, including frontal and occipital regions. Previous studies did not find a significant effect of 3 weeks’ SSRI on emotion processing in cortical regions^24,47^, perhaps explained by their relatively smaller sample sizes. The frontal cortex has been identified as critical for face processing^5^ and argued to exert top–down cognitive control of the amygdala^63,64^. This fronto-amygdala functional brain network is involved in regulating emotions^65^, and found to be compromised in MDD^66^. The observed SSRI-induced modulation of emotion processing in frontal regions may relate to the clinical efficacy of SSRIs. Region-specific effects of sub-chronic SSRIs on emotion processing could stem from region-specific molecular changes in 5-HT tonus; a previous study in healthy individuals from our lab reported increased 5-HT tonus in some, but not all, regions following sub-chronic SSRI intake^67^. Future studies using positron emission tomography (PET) could identify the serotonergic receptors involved in this neuro-modulation of emotional face processing.

In contrast to our finding of a decreased brain response to angry and fearful faces, we observed significantly higher responses to neutral faces in frontal regions compared to placebo. This finding indicates that SSRI intake over a clinically relevant time period does not reduce brain responses to facial expressions per se but rather specifically to negative emotions (i.e., fear and anger), a valence-specific effect that may be associated with SSRIs' antidepressant properties^34^. As the modulatory effect of 5-HT seems to depend on emotional valence, we recommend future studies investigating serotonergic modulation of face processing to explore emotional face valence separately instead of collapsing them into an overall outcome of face processing, as done in some previous studies^38,39,47^.

In sum, our findings suggest that 3–5 weeks of SSRI intake valence specifically reduces brain response to negative faces, but in brain regions outside the amygdala, particularly in frontal regions. These results provide unique insight into possible mechanisms behind the clinical efficacy of SSRIs. However, we did not evaluate brain responses to positive valence faces (e.g., happy faces); future studies of sub-chronic SSRI effects also examining happy faces would provide greater insight into possible valence-specific effects.

In post hoc analyses, we observed that effects on a voxel-wise level showed a similar pattern to those observed on a regional level, e.g., lower response to negative faces in frontal and occipital brain regions. However, no clusters remained statistically significant evaluating whole-brain effects nor focusing on the amygdala after correcting for multiple comparisons. We suspect that the apparent discrepancy between the effects of SSRI on a voxel-wise compared to regional level may stem from relatively limited statistical power for the voxel-wise analyses.

### Methodological consideration

This study is not without limitations. The emotional faces paradigms have been shown to have low within-subject test–retest reliability^68,69^. Although this mitigates the lack of a baseline scan in estimating drug effects, it nevertheless represents a noise source that may challenge our capacity to detect group differences.

Five participants dropped out due to side effects, of whom four received SSRI. Assuming that this group had an overall greater psychoactive response to SSRI, these missing data may have biased our results towards underestimating SSRI effects on amygdala reactivity.

In this study, we examined only brain responses per se. Future studies should investigate alternative modelling of these data, e.g., brain connectivity, which may capture relevant neural correlates associated with SSRI intervention^65^.

Participants receiving SSRI guessed their group assignment correctly at chance level, but the frequency of guessing SSRI group assignment was significantly higher than those in the placebo group (i.e., 16%). This contrasts with our previous study in healthy males with the SSRI fluoxetine, where nearly all participants guessed that they received a placebo^70^. The reason for the observed group discrepancy and whether it reflects an influential confound is not apparent, but its presence is notable. It is possible that perceptual differences influencing participants’ guesses could be similarly associated with our observed exploratory group differences in frontal and occipital brain responses.

Our study population consisted of a North European sample of younger age, which limits our results' generalisability to other populations. Whether the results can be replicated in more diverse populations should be investigated in future studies.

In this study, we evaluated brain responses and related mood changes at a single time point. Future studies evaluating brain responses at multiple time points during SSRI intake could probe the trajectory of the drug’s effects on brain response to emotional material, which may provide a more nuanced perspective on associated brain effects.

## Conclusion

We did not find evidence that 3–5 weeks of intake of SSRI significantly affected amygdala response to threat-related or neutral faces in a large cohort of healthy individuals. However, whole-brain analyses revealed significant effects in the frontal and occipital regions. These results implicate serotonergic modulation of top–down emotional processing, a modulation that may be involved in SSRIs’ clinical efficacy.

### Supplementary Information

Supplementary Information 1.

Supplementary Table S1.

Supplementary Table S2.

Supplementary Table S3.

Supplementary Table S4.

Supplementary Table S5.

## Data Availability

The data that support the findings of this study are available from the Cimbi database managed by Peter S. Jensen^71^, but restrictions apply to the availability of these data, which were used under license for the current study, and so are not publicly available. Data are however available from Peter S. Jensen upon reasonable request and with permission of steering members of the Cimbi database who can be contacted through info@nru.dk.
