# Supplementary material for: Functional brain responses to emotional faces after three to five weeks of intake of escitalopram in healthy individuals: a double-blind, placebo-controlled randomised study
Source: Sci Rep. 2024 Feb 7;14:3149. doi: 10.1038/s41598-024-51448-2 (PMC10850508; doi:10.1038/s41598-024-51448-2)
Supplement: Supplementary file 1 — Supplementary Information 1. [file 41598_2024_51448_MOESM1_ESM.docx]

**SUPPLEMENTARY MATERIAL**

**Functional brain responses to emotional faces after three to five weeks of intake of escitalopram in healthy individuals: A double-blind, placebo-controlled randomised study**

Sophia Armand, PhD,^1,2^ Christelle Langley, PhD,^3^ Annette Johansen, MD,^1^ Brice Ozenne, PhD,^1,4^ Oliver Overgaard-Hansen, BSc,^1^ Kristian Larsen,^1,5^ Peter S. Jensen, MSc,^1^ Gitte M. Knudsen, DMSc^1,5^ Barbara J. Sahakian, DSc,^3^ Dea S. Stenbæk, PhD,*#^1,2^ & Patrick M. Fisher, PhD#^1,6^

#The authors contributed equally to the manuscript

^1^Neurobiology Research Unit, Copenhagen University Hospital Rigshospitalet, Denmark

^2^ Department of Psychology, University of Copenhagen, Denmark

^3^Department of Psychiatry, University of Cambridge, Cambridge, United Kingdom.

^4^Department of Public Health, Section of Biostatistics, University of Copenhagen, Denmark

^5^Department of Clinical Medicine, University of Copenhagen, Denmark

^6^Department of Drug Design and Pharmacology, University of Copenhagen, Denmark

**Corresponding author:**

Dea Siggaard Stenbæk, Associate Professor

Neurobiology Research Unit, Rigshospitalet, 6-8 Inge Lehmanns Vej, building 8057**,** DK-2100 Copenhagen O. Ph: +45 20 83 4838, mail: [dea@nru.dk](mailto:dea@nru.dk), fax: +45 35 45 67 13

Words in manuscript: 2894 (not including abstract, methods, acknowledgements, author contribution, source of funding and conflicts of interest, data availability statement, references, and figure/table legends)

Number of appendices in the supplementary materials: 4

Number of tables in the supplementary materials: 5

**Appendix 1**

Inclusion of participants and missing data

From an initial group of 73 included volunteers who started the intervention, five participants dropped out due to self-reported side effects (of which four participants received SSRI and one received placebo), and two participants did not complete the follow-up assessment due to sickness or pain during MRI-scanning. One participant was excluded following an incidental finding of severe brain atrophy on MRI.

One participant was excluded based on poor behavioural task performance (i.e., accuracy and reaction time), indicating no attention to the emotional face task.

**Appendix 2**

Exclusion criteria

The exclusion criteria were: 1) Current or former primary psychiatric disorder as classified in DSM-V or WHO ICD-11, 2) Current or former neurological disease or severe somatic disease, 3) Head injury or concussion resulting in loss of consciousness for more than two min., 4) Current use of psychoactive medication, 5) Drug use other than tobacco and alcohol within the last 30 days, 6) Alcohol or drug abuse, 7) Use of cannabis more than 50 times, 8) Use of illegal psychoactive drugs more than ten times for each drug, 9) Use of any drugs likely to influence the test results, 10) Nicotine addiction, 11) Allergy to the ingredients in the administered drug, 12) Abnormal ECG, such as prolonged QT syndrome, 13) Dizziness when changing from supine to upright position (e.g. postural orthostatic tachycardia syndrome), 14) Mild hypotension (blood pressure below 100/70 mmHg) or hypertension (blood pressure above 140/90 mmHg), 15) Contraindications for MRI such as a pacemaker or soft-tissue metal, 16) Pregnancy or breast-feeding, 17) Current or past learning disability, 18) Non-fluent in Danish, 19) Pronounced visual or auditory impairments, 20) Severe physical impairments affecting eyesight or motor performance.

**Appendix 3**

### Magnetic Resonance Imaging

At the end of the intervention period, participants completed an MRI scan on a 3T Siemens Magnetom Prisma scanner (Erlangen, Germany) using a 32-channel head coil. We acquired a high-resolution, whole-brain, T1-weighted MPRAGE structural scan (inversion time = 972 ms, repetition time (TR) = 2000 ms, echo time (TE) = 2.58 ms, flip angle = 8°, in-plane matrix = 256x256 mm, in-plane resolution = 0.9x0.9 mm, 224 slices, slice thickness = 0.9 mm), followed by BOLD fMRI scans acquired during the emotional faces paradigm using a T2*-weighted gradient echo-planar imaging (EPI) sequence (TR = 2000 ms, TE = 30 ms, flip angle = 70^o^, in-plane matrix = 76x76 mm, in-plane resolution=3x3 mm, 35 slices (thickness = 3.0 mm, gap between slices= 0.6 mm), number of volumes acquired = 195).We acquired a gradient field map to ameliorate spatial distortions in the BOLD fMRI acquisition.

**Appendix 4**

Pre-processing of fMRI data

MRI images were pre-processed and analysed in Statistical Parametric Mapping 12 (SPM12) (https://www.fil.ion.ucl.ac.uk/ spm/software/spm12/). Single-subject functional volumes were corrected for slice-timing, unwarped, realigned to a subject-specific mean functional image, and smoothed using a 4 mm FWHM Gaussian filter. Functional images were kept in subject space for analysis because we focused on regional effects and sought to limit image modulation (e.g., interpolation). The Automatic Anatomical Label (AAL) atlas was used to define regions of interest (ROIs).^56^ The T1-weighted structural image was co-registered with the functional images and then normalised into Montreal Neurological Institute (MNI) standard space. The subject-specific inverse deformation map was applied to warp the AAL atlas into subject space and then resliced to match the functional images (both procedures applied nearest-neighbour interpolation to retain region labels). Artefact Detection Tool (ART; https://www.nitrc.org/projects/artifact_detect/) was applied to identify individual functional volumes with excess motion (>2 mm) and/or signal variability (>4 SD), which were censored from task-related analyses.

For voxel-wise analyses, single-subject co-registered T1-weighted structural images were normalised into MNI space and the associated forward deformation map was applied to the functional images. Functional images were then smoothed with 6 mm FWHM Gaussian filter. ART was applied to the processed images and used to censor outlier volumes from task-related analyses as described above.

| **Estimated model coefficients when regressing amygdala response to angry faces** | | | | |
| --- | --- | --- | --- | --- |
|  |  |  |  |  |
|  |  |  |  |  |
|  | Estimate | SE | p_unc_ | p_FWER_ |
| Constant | 0.65 | 0.47 | 0.18 |  |
| SSRI | -0.10 | 0.20 | 0.63 | 1.000 |
| Age | -0.01 | 0.02 | 0.93 | 1.000 |
| Sex | -0.01 | 0.02 | 0.55 | 1.000 |
| **Estimated model coefficients when regressing amygdala response to fearful faces** | | | | |
|  |  |  |  |  |
|  |  |  |  |  |
|  | Estimate | SE | p_unc_ | p_FWER_ |
| Constant | 0.13 | 0.28 | 0.65 |  |
| SSRI | 0.05 | 0.12 | 0.68 | 1.000 |
| Age | 0.00 | 0.01 | 0.86 | 1.000 |
| Sex | 0.33 | 0.12 | 0.01 | 0.023 |
| **Estimated model coefficients when regressing amygdala response to neutral faces** | | | | |
|  |  |  |  |  |
|  |  |  |  |  |
|  | Estimate | SE | p_unc_ | p_FWER_ |
| Constant | 0.65 | 0.40 | 0.11 |  |
| SSRI | 0.09 | 0.17 | 0.59 | 1.000 |
| Age | -0.01 | 0.01 | 0.51 | 1.000 |
| Sex | -0.30 | 0.17 | 0.08 | 0.255 |
| **Table A.** The effect of SSRI (n=32), relative to placebo (n=32), on amygdala response to angry, fearful, and neutral faces (contrasted with geometric shapes) during BOLD fMRI, was assessed using mixed linear effect models. Covariates included age and sex. Sex is the marginal effect of men relative to women. The estimate is the mean differences between groups. Abbreviations: SE = standard error for unstandardised beta, Punc = unadjusted significance level, 95% CI = 95% confidence interval, SSRI = selective serotonin reuptake inhibitors. All p-values were corrected for family-wise multiple testing using the Bonferroni method (i.e. all p-values multiplied by three), displayed under PFWER. | | | | |
|  |  |  |  |  |
|  |  |  |  |  |
|  |  |  |  |  |
|  |  |  |  |  |
|  |  |  |  |  |
|  |  |  |  |  |
|  |  |  |  |  |
|  |  |  |  |  |
|  |  |  |  |  |

| **Correlation between amygdala response to angry faces and mood disturbances** | | | | | |
| --- | --- | --- | --- | --- | --- |
|  |  |  |  |  |  |
|  | Estimate | SE | p_unc_ | 95% CI | |
| Placebo | 0.24 | 0.19 | 0.211 | -0.15 | 0.56 |
| SSRI | -0.12 | 0.19 | 0.542 | -0.47 | 0.27 |
| **Correlation between amygdala response to fearful faces and mood disturbances** | | | | | |
|  |  |  |  |  |  |
|  | Estimate | SE | p_unc_ | 95% CI | |
| Placebo | -0.32 | 0.19 | 0.092 | -0.621 | 0.06 |
| SSRI | -0.03 | 0.19 | 0.886 | -0.40 | 0.35 |
| **Correlation between amygdala response to neutral faces and mood disturbances** | | | | | |
|  |  |  |  |  |  |
|  | Estimate | SE | p_unc_ | 95% CI | |
| Placebo | 0.41 | 0.19 | 0.033 | 0.04 | 0.68 |
| SSRI | 0.11 | 0.19 | 0.568 | -0.28 | 0.47 |
| **Table B.** Partial correlations between amygdala response to angry/fearful/neutral faces and negative mood states adjusted for covariates age and sex. The amygdala response to angry, fearful, and neutral faces (contrasted with geometric shapes) are assessed using BOLD fMRI. Negative mood states are measured with the self-report questionnaire Profile of Mood States (POMS), with higher values indicating more negative mood. The estimate is rho. SE = standard error, Punc = unadjusted significance level, 95% CI = 95% confidence interval, SSRI = selective serotonin reuptake inhibitors, which was administered 3-5 weeks. | | | | | |
|  |  |  |  |  |  |
|  |  |  |  |  |  |
|  |  |  |  |  |  |
|  |  |  |  |  |  |
|  |  |  |  |  |  |
|  |  |  |  |  |  |
|  |  |  |  |  |  |
|  |  |  |  |  |  |
